# A Sudden Onset of Severe Thrombocytopenia While Using Evolocumab

**DOI:** 10.1155/2020/3281626

**Published:** 2020-03-26

**Authors:** Ikuo Inoue, Yasuhiro Takenaka, Yoshitora Kin, Satoshi Yamazaki, Yuichi Ikegami, Daigo Saito, Akira Shimada

**Affiliations:** ^1^Department of Endocrinology and Diabetes, Faculty of Medicine, Saitama Medical University, 38 Morohongo, Moroyama, Iruma, Saitama 350-0495, Japan; ^2^Department of Physiology, Graduate School of Medicine, Nippon Medical School, 1-25-16 Nezu, Bunkyo, Tokyo 113-0031, Japan; ^3^Department of Internal Medicine, Fukaya Red Cross Hospital, 5-8-1 Kamishibatyohnishi, Fukaya, Saitama 366-0052, Japan; ^4^Kumagaya-geka Hospital, 3811-1 Sayata, Kumagaya, Saitama 360-0023, Japan

## Abstract

A 72-year-old man with a 10-year history of coronary heart disease started evolocumab treatment once a month after developing excess myalgia due to therapy with a 3-hydroxy-methylglutaryl CoA reductase inhibitor. No side effects such as myalgia symptoms had been reported during the first 14 months of evolocumab treatment; however, he suddenly presented with acute severe thrombocytopenia following the 14th treatment. His platelet count continued to decrease to a nadir of 1,000/*μ*L. His platelet-associated immunoglobulin G level had elevated to 790 ng/10^7^ cells. He started receiving a combination of steroid therapy, high-dose immunoglobulin therapy, and platelet transfusions, but the first-line therapy was ineffective. He was subsequently treated with a thrombopoietin receptor agonist, and his platelet count recovered to 250,000/*μ*L.

## 1. Introduction

3-Hydroxy-methylglutaryl CoA (HMG-CoA) reductase inhibitor (statin) therapy reduces coronary heart disease (CHD) events through decreasing low-density lipoprotein-cholesterol (LDL-C) levels. However, between 5% and 10% of statin users develop statin intolerance. Statin intolerance refers to an inability to use statins because of myalgia symptoms or elevated serum creatine kinase levels. It has been reported that patients with statin intolerance may have an increased risk of CHD events [1]; therefore, new and efficient LDL-C-lowering drugs via a different mechanism from statins are necessary.

Recently, proprotein convertase subtilisin/kexin type 9 (PCSK9) inhibitors have safely been administered to patients worldwide. Evolocumab, a PCSK9 inhibitor, became available in Japan in 2016, and 3 months later, alirocumab was also made available. Evolocumab [2] and alirocumab [3] are human immunoglobulin G2 and G1 monoclonal antibodies, respectively, against PCSK9 expressed by hepatocytes. Evolocumab or alirocumab therapy reduces CHD events through decreasing LDL-C levels, as seen in statin therapy. Evolocumab and alirocumab have been reported to be effective for patients with CHD without severe side effects. Mild adverse effects, such as an allergic reaction at the injection site, have been observed with the use of evolocumab or alirocumab. There has been no reported incidence of serious systemic hypersensitivity reactions such as anaphylaxis [4]. Moreover, during the clinical use of evolocumab or alirocumab, significant hematologic changes such as thrombocytopenia have not been reported so far. Furthermore, even in large-scale intervention studies, the incidence of side effects secondary to evolocumab or alirocumab use during follow-up has been reported to be similar to that of the placebo [4]. Here, we describe a rare case of thrombocytopenia that developed during the use of evolocumab.

## 2. Case report

A 72-year-old man with a 10-year history of CHD and hyperlipidemia started evolocumab therapy once a month after he had developed excess myalgia due to statin treatment. His hematological laboratory data were normal. His CHD diagnosis had been supported by coronary computed tomographic angiography findings. He had complained of chest pain at various times between the age of 65 and 70 years. He had a family history of CHD.

**Figure 1:**
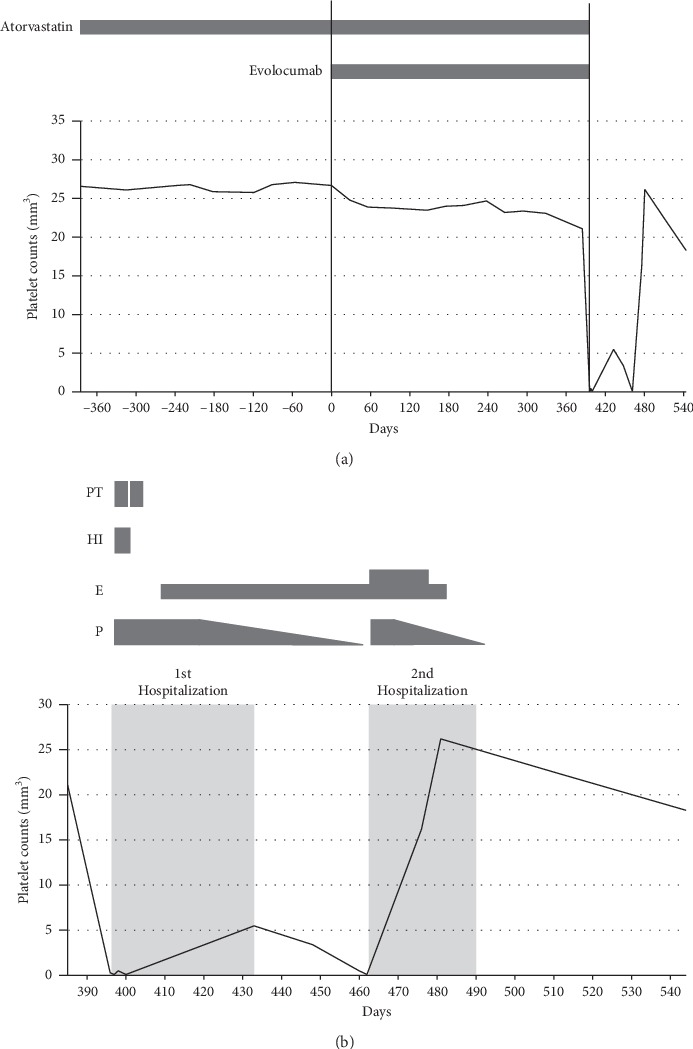
Patient's platelet counts relative to the time courses of his treatment before (a) and after (b) hospitalization. In (a) and (b), the *Y*-axis and the *X*-axis represent platelet counts and the number of days before and after the first treatment of evolocumab, respectively. Day 0 indicates the initial day of evolocumab therapy. The periods of atorvastatin and evolocumab treatment are indicated above the graph in (a). PT, platelet transfusions (two injections); HI, high-dose immunoglobulin (25 g per day for 5 consecutive days); E, eltrombopag (thin line, 12.5 mg; thick line, 25 mg per day, respectively); P, prednisone (starting with 1 mg/kg).

The patient had no thrombocytopenia during his monthly combined treatment with evolocumab (140 mg) and low-dose atorvastatin (2.5 mg per day). His platelet count was largely unchanged (225,000–260,000/*μ*L) for 14 months from the first evolocumab administration. On the 13th and just prior to the 14th treatment with evolocumab, his platelet count was 211,000 and 210,000/*μ*L, respectively; however, it suddenly decreased to 1,000/*μ*L on day 12 following his 14th treatment with evolocumab (Figure 1(a)), and he presented with symptoms of acute severe thrombocytopenia. A physical examination revealed nonpalpable petechial purpura over his face, extending to the neck, the front and back of the trunk, and both limbs. In addition, sudden-onset gingival bleeding and epistaxis were observed. He also presented with nasal bleeding, ocular hyperemia, and hemorrhages in the buccal mucosa. He was immediately admitted to a hospital.

The patient's platelet count at admission was 3,000/*μ*L. His platelet count continued to decrease over 12 days following evolocumab discontinuation to a nadir of 1,000/*μ*L, and he had slight anemia. The patient's reticulocyte count, serum iron level, and direct antiglobulin test results were normal (data not shown). Although his red blood cell count, hemoglobin, and hematocrit values slightly decreased from 3.39 to 3.27 × 10^4^ cells/*μ*L, from 11.5 to 11.1 g/dL, and from 34.8 to 33.8%, respectively, these changes were not significant. His white blood cell count slightly increased from 3,430 to 4,100/*μ*L. A bone marrow aspiration examination revealed neither increase in the number of megakaryocytes nor any morphologic abnormalities. A cytogenetic study of megakaryocytes showed normal karyotype and absence of chromosomal rearrangements such as translocations and deletions. He tested negative for *Helicobacter pylori* infection. Inflammatory workup was performed for the negative erythrocyte sedimentation rate, anti-ribonucleoprotein antibody, anti-double-stranded DNA antibody, rheumatoid factor, and complement levels. Cytomegalovirus, human immunodeficiency virus, Epstein–Barr virus, immunoglobulin G (IgG), and IgM were not detected in the patient. Spleen size was measured using abdominal echo examination. His spleen was 9 cm long and 4 cm wide, which was in the normal range. His platelet-associated immunoglobulin G (PAIgG) level was elevated at 790 ng/10^7^ cells. Therefore, the patient was newly diagnosed with immune thrombocytopenia.

The patient started a combination of steroid therapy, high-dose immunoglobulin therapy, and platelet transfusions (Figure 1(b)). After platelet transfusion therapy, his platelet count increased after 1 h, but was decreased again by 4 h. He was treated with prednisone (1 mg/kg/day for 3 weeks), but this resulted in no improvement of the thrombocytopenia and the initial therapy was ineffective. Owing to persistent thrombocytopenia, he was started on eltrombopag, which is a small molecule thrombopoietin receptor agonist, at a low starting dose of 12.5 mg. Thereafter, in addition to 25 mg eltrombopag, we administered a daily dose of 60 mg prednisone to achieve a >250,000/*µ*l platelet count. Three months later, his platelet count had returned to normal and remained at approximately 200,000/*μ*L during the 2-month follow-up period post-initiation of eltrombopag (Figure 1(b)). No bleeding complications were observed post-discharge. Currently, without steroid administration, his platelet count is stable at approximately 183,000/*μ*L.

## 3. Discussion

Here, we describe a rare complication of severe thrombocytopenia that occurred while using evolocumab. There are two possible mechanisms of thrombocytopenia: decreased platelet production and increased platelet destruction. When a platelet count decreases, megakaryocytes usually increase in number as a compensatory action. However, in our case, a bone marrow aspiration examination revealed no increase in the number of megakaryocytes. Therefore, in the present case, we should consider both platelet destruction, as well as incomplete maturation of megakaryocytes, as the potential mechanism leading to thrombocytopenia.

The patient was diagnosed with acute ITP instead of drug-induced immune thrombocytopenia (DITP), although our assays were inconclusive. In general, DITP occurs 1 to 2 weeks after the initial administration of a particular drug with a sudden decrease in platelet counts to <100,000/*μ*L [5, 6]. This causes clinically significant hemorrhage in patients, and deaths have been reported [7]. In our case, the patient's platelet counts were within the normal range from the first to the 14th evolocumab administration, but suddenly dropped 12 days after the 14th administration (Figure 1(a)). In some cases, DITP can occur following intermittent use of a drug such as quinine, the oldest reported cause of thrombocytopenia, for an extended period of time [5, 7, 8]. Thus, we were unable to exclude the possibility that evolocumab was relevant to the development of drug-induced and immune-mediated destruction of platelets in the patient. PAIgG level was elevated during thrombocytopenia, which may have largely bound to platelets and induced platelet aggregation or lysis, resulting in fewer platelets. Another possibility was that evolocumab impaired the maturation of megakaryocytes to produce platelets, although they were normal in number. Human megakaryocytes express PCSK9 transcripts at low level [9]. Thus, evolocumab potentially interacted with a part of the PCSK9 on the surface of megakaryocytes, which might have, in turn, caused disturbances in the signaling pathways of megakaryocyte differentiation.

To date, there have been no reported cases of severe thrombocytopenia involving patients receiving evolocumab. Barale et al. reported reduced platelet reactivity to agonists, such as adenosine diphosphate and collagen, in patients with familial hypercholesterolemia after 8-week treatment with evolocumab or alirocumab [10]. Although the presence of significant changes in the platelet count were not described in their report, senile or damaged platelets with decreased function may be quickly removed from the blood, resulting in fewer platelets.

Plasma PCSK9 levels have been shown to positively correlate with the platelet count in patients with stable coronary artery disease with unknown physiological mechanisms [11]. In our case, although the plasma PCSK9 level was not measured before or after the onset of thrombocytopenia, it is plausible that the administered evolocumab formed a drug-target complex with PCSK9 to block its function, thereby facilitating degradation and reducing the plasma PCSK9 levels. A prompt reduction in the plasma PCSK9 level via interaction with the drug may have led to a decreased platelet count. The relationship between the plasma PCSK9 level and the platelet count in patients with hypercholesterolemia needs to be investigated.

Significant hematologic changes, such as thrombocytopenia, have not been reported with the clinical use of evolocumab or alirocumab. However, hematologic parameters, especially changes in platelet count, should be carefully monitored during PCSK9 inhibitor therapy. We believe that our case report provides a new insight into the pharmacological aspects of lipid-lowering drugs in patients with coronary heart disease.

## Supplementary Materials

Supplementary Materials Supplementary Figure 1: speculative schema of the mechanism of evolocumab-dependent platelet destruction through immune mechanisms. PCSK9-evolocumab complex containing IgG interacting with the platelet Fc receptors (a) and evolocumab-platelet membrane complex harboring neoantigenic determinants where excess evolocumab attaches onto the platelet membrane (b). In the former case, IgG directed against the PCSK9-evolocumab complex as the antigen was generated, and the complex bound to the platelet Fc receptor through the antibodies. PAIgG, platelet-associated immunoglobulin G.

## References

[1] Serban M.-C., Colantonio L. D., Manthripragada A. D. (2017). Statin intolerance and risk of coronary heart events and all-cause mortality following myocardial infarction. *Journal of the American College of Cardiology*.

[2] Fala L. (2016). Repatha (evolocumab): second PCSK9 inhibitor approved by the FDA for patients with familial hypercholesterolemia. *Am Health Drug Benefits*.

[3] Manniello M., Pisano M. (2016). Alirocumab (Praluent): First in the new class of PCSK9 inhibitors. *P T*.

[4] Hirayama A., Yamashita S., Inomata H. (2017). One-year efficacy and safety of evolocumab in Japanese patients―A pooled analysis from the open-label extension OSLER studies ―. *Circulation Journal*.

[5] George J. N., Aster R. H. (2009). Drug-induced Thrombocytopenia: Pathogenesis, Evaluation, and Management. *Hematology*.

[6] Ali N., Auerbach H. E. (2017). New-onset acute thrombocytopenia in hospitalized patients: pathophysiology and diagnostic approach. *Journal of Community Hospital Internal Medicine Perspectives*.

[7] Aster R. H., Bougie D. W. (2007). Drug-induced immune thrombocytopenia. *New England Journal of Medicine*.

[8] Reddy J. C., Shuman M. A., Aster R. H. (2004). Quinine/quinidine-induced thrombocytopenia: a great imitator. *Archives of Internal Medicine*.

[9] Paciullo F., Momi S., Gresele P. (2019). PCSK9 in haemostasis and thrombosis: possible pleiotropic effects of PCSK9 inhibitors in cardiovascular prevention. *Thrombosis and Haemostasis*.

[10] Barale C., Bonomo K., Noto F. (2017). Effects of PCSK9 inhibitors on platelet function in adults with hypercholesterolemia. *Atherosclerosis*.

[11] Li S., Zhu C.-G., Guo Y.-L. (2015). The relationship between the plasma PCSK9 levels and platelet indices in patients with stable coronary artery disease. *Journal of Atherosclerosis and Thrombosis*.

