# Supplementary material for: A Sudden Onset of Severe Thrombocytopenia While Using Evolocumab
Source: Case Rep Hematol. 2020 Mar 26;2020:3281626. doi: 10.1155/2020/3281626 (PMC7136763; doi:10.1155/2020/3281626)
Supplement: Supplementary Materials — Supplementary Figure 1: speculative schema of the mechanism of evolocumab-dependent platelet destruction through immune mechanisms. PCSK9-evolocumab complex containing IgG interacting with the platelet Fc receptors (a) and evolocumab-platelet membrane complex harboring neoantigenic determinants where excess evolocumab attaches onto the platelet membrane (b). In the former case, IgG directed against the PCSK9-evolocumab complex as the antigen was generated, and the complex bound to the platelet Fc receptor through the antibodies. PAIgG, platelet-associated immunoglobulin G. [file 3281626.f1.pdf]

Supplementary Figure 1

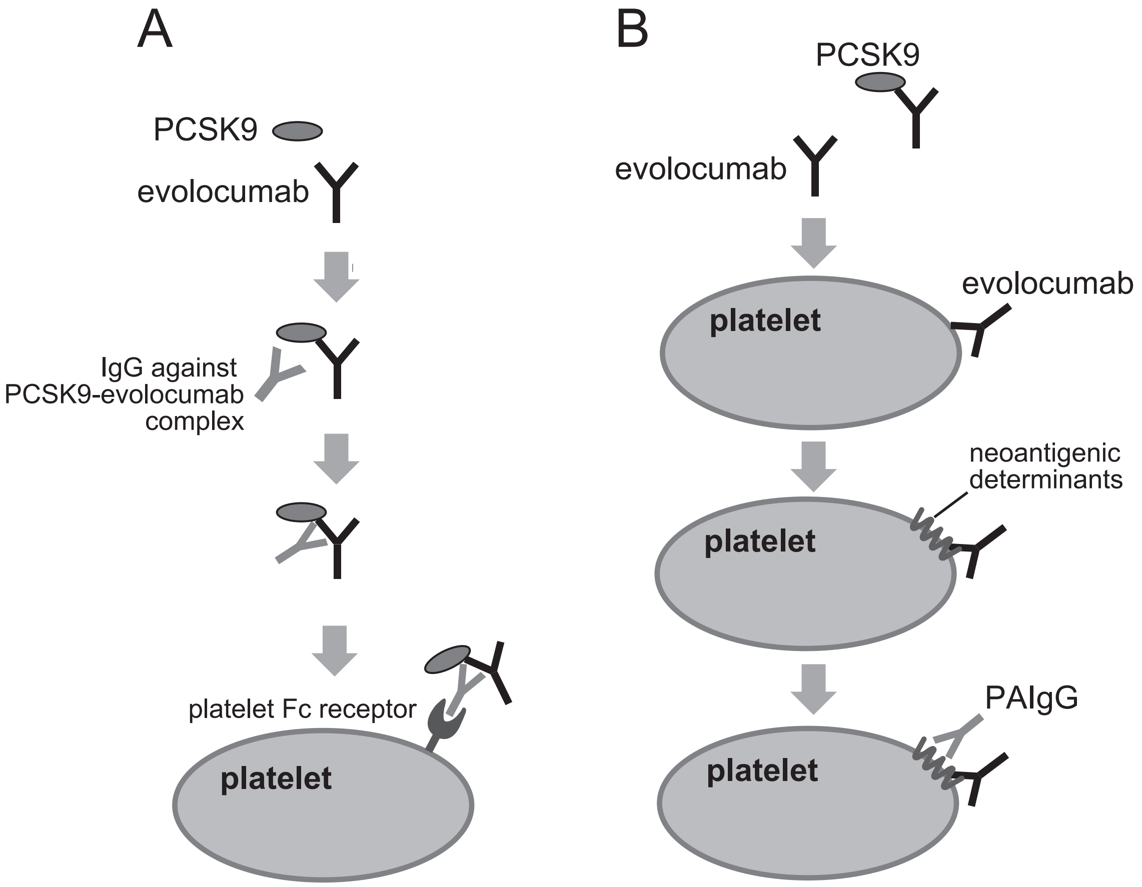

Speculative schema of the mechanism of evolocumab-dependent platelet destruction through immune mechanisms. PCSK9-evolocumab complex containing IgG interacting with the platelet Fc receptors (A) and evolocumab-platelet membrane complex harboring neoantigenic determinants where excess evolocumab attaches onto the platelet membrane (B). In the former case, IgG directed against the PCSK9-evolocumab complex as the antigen was generated, and the complex bound to the platelet Fc receptor through the antibodies. PAIgG, platelet-associated immunoglobulin G.
